# A real-time PCR screening assay for the universal detection of lumpy skin disease virus DNA

**DOI:** 10.1186/s13104-019-4412-z

**Published:** 2019-07-01

**Authors:** Sprygin Alexander, Byadovskaya Olga, Kononova Svetlana, Zakharov Valeriy, Pestova Yana, Prutnikov Pavel, Kononov Aleksandr

**Affiliations:** Federal Center for Animal Health, Vladimir, 600901 Russia

**Keywords:** Lumpy skin disease, Real-time PCR, Screening, Vaccine strain, Field strain, Recombinant strain

## Abstract

**Objective:**

The resurgence of lumpy skin disease virus isolates of different genotypic natures abolishes the accuracy of assays that target either vaccine or field strain genome. The aim of the present study was to develop a universal real-time PCR assay using TaqMan chemistry to cover field, vaccine, and recombinant strains of lumpy skin disease virus isolates.

**Results:**

The PCR assay was designed based on a LSDV044 target region that offers a unique identification locus to facilitate the sensitive and specific detection of all isolates known to date. The efficiency of amplification, determined over five orders of magnitude, was 93%, with the standard deviation remaining in the range of 0.11–0.23. Evaluation of the assay repeatability on three different days revealed that the inter-run variability ranged from 0.83 to 1.22 over five repetitions across three runs. This new screening assay is proposed as a fast, efficient, and sensitive tool that can be employed in the basic or applied surveillance studies regardless of the genotype. Moreover, the assay can be used for the routine laboratory testing of animal samples during eradication programs for lumpy skin disease.

## Introduction

Lumpy skin disease virus (LSDV) is the etiologic agent of lumpy skin disease in cattle, which inflicts high morbidity, leading to considerable economic losses in Africa, Asia, the Middle East, Russia, and Europe [1–4]. LSDV is an enveloped capripoxvirus from the *Poxviridae* family. Its genome comprises double-stranded DNA and is approximately 150,000 base pairs (bp) long [5].

Once lumpy skin disease is suspected among susceptible animals, timely diagnosis plays a crucial role in the implementation of control measures to save expenses and contain the disease. Disease prevention should be implemented via vaccination with sheep pox- and attenuated LSDV-based vaccines [6, 7].

The heavy use of live attenuated vaccines against LSDV can cause co-infection with a vaccine and field strain in a host [8]. The recent emergence of a recombinant vaccine strain eliminated the efficacy of assays capable of differentiating infected from vaccinated animals (DIVA) [9, 10] and calls for updated assays that provide more specificity at least at the species level. Positive results can be followed up by sequencing to discover the genetic background of an obtained strain.

Here, we developed a real-time PCR assay based on the unique site in LSD044 for the universal detection of DNA from field, vaccine, and recombinant strains of LSDV.

## Main text

### Methods

#### Primers and probe

**Fig. 1:**
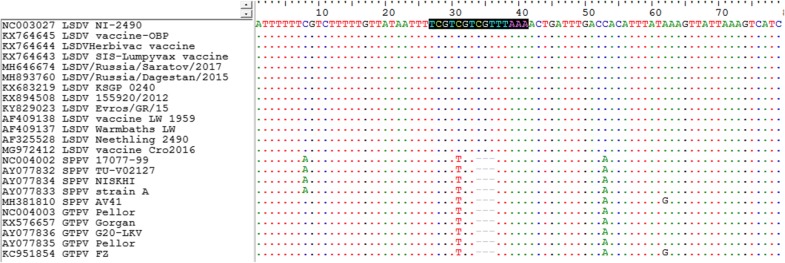
Nucleotide alignment of the probe targeted gene sequence of LSDVs

The assay was designed based on a target that offers a unique identification locus to facilitate the sensitive and specific detection of LSDV. For designing candidate primers and probes, complete genome nucleotide sequences from all known LSDV strains as well as sheep pox viruses (SPPV) and goat pox viruses (GTPV) were recovered from GenBank and aligned using Bioedit to select the target region (Fig. 1). Overall, 23 capripoxvirus complete genome sequences were retrieved (KX683219 KSGP 0240, KY829023 Evros/GR/15, AF409137 Neethling Warmbaths LW, AF325528 Neethling 2490, KX894508 LSDV 155920/2012, NC003027 LSDV NI-2490, MH646674 LSDV Saraov/2017, MH893760 LSDV Dagestan/2015, KX764643 SIS-Lumpyvax vaccine, KX764644 Neethling-Herbivac vaccine, AF409138 Neethling vaccine LW 1959, KX764645 Neethling-LSD vaccine-OBP, MG972412 LSDV vaccine Cro2016, KX576657 Gorgan, KC951854 FZ, AY077836 G20-LKV, AY077835 Pellor, NC004003 GTPV Pellor, AY077833 Sheeppox virus A, AY077832 Sheeppox virus 10,700–99 strain TU-V02127, MG000156 Sheeppox virus NISKHI, MH381810 SPPV AV41, NC004002 SPPV 17077–99).

The primers flanked a conserved 151-bp region of LSD044 that is found among LSDV strains: zdf4ln (forward primer) CAA + AAA + CAA + TCG + TAAC + TAATCCA, zdr4ln (reverse primer) TG + GAGTTTTTA + TG + TCATCGTC, and (Taqman® probe) zdpro4ln1 TC + GT + CGT + CG + TT + TAA + AACTGA. The Taqman probe was labeled with 6-carboxy fluorescein (FAM), the reporter dye at the 5′-end and he Black Hole Quencher-1 (BHQ-1) at the 3′-end) According to the alignment, all currently sequenced sheep and goat pox strains contain a 3-bp deletion in the probe targeting site (Fig. 1). Selected primers were synthesized by Syntol (Moscow, Russia) and modified with Locked Nucleic Acid (LNA) bases (on the right side of the plus sign). The Basic Local Alignment Search Tool (BLAST) analysis was conducted to test primers against the currently deposited LSDV strains in GenBank. Reference and field strain samples used in the study are presented in Table 1.

**Table 1:** Capripoxvirus strain DNA used in the study

Sample	Mean Ct ± SD
LSDV Russia, 2015	29.99 ± 0.27
LSDV Russia, 2016	30.75 ± 0.45
LSDV Russia, 2017	31.55 ± 0.36
LSDV Abkhazia, 2017	31.76 ± 0.41
LSDV Kazakhstan, 2016	32.81 ± 0.49
LSDV Ethiopia, 1995	24.16 ± 0.11
LSDV unknown origin and year	25.8 ± 0.27
LSDV Massalamia	32.21 ± 0.21
LSDV Bulgaria*	33.85 ± 0.34
vaccine LSDV Onderspoort vaccine strain	29.17 ± 0.19
LSDV Lumpyvax	30.55 ± 0.35
Neethling LSDV *	30.01 ± 0.11
vaccine-like LSDV Russia, 2017	29.39 ± 0.12
vaccine-like LSDV Russia, 2017	31.00 ± 0.24
vaccine-like LSDV Russia, 2017	31.55 ± 0.39
recombinant LSDV strain, 2017	29.99 ± 0.28
SPPV Afghanistan, 2003	no Ct
SPPV Russia, 2015	no Ct
SPPV Russia, 2016	no Ct
SSPV Niskhi	no Ct
SSPV Morocco	no Ct
SPPV Arbel*	no Ct
SSPV (unknown origin)*	no Ct
GTPV Russia, 2003	no Ct
GTPV Gorgan	no Ct
GTPV Oman	no Ct

*Positive samples were supplied by Sciensano (Belgium)

#### DNA extraction

Total viral DNA was extracted from the culture samples or sera of infected animals using the QIAamp DNA Mini Kit (Qiagen, Germany), as per the manufacturer’s instructions.

#### PCR assay

PCR was conducted on a Rotor-Gene Q thermal cycler (Qiagen, Germany) using the following program: 95 °C for 5 min, followed by 40 cycles of 95 °C for 15 s, and 60 °C for 60 s. Each 25-μL PCR reaction comprised 5 μL of DNA, 5 μL of 5 × PCR buffer (Promega, USA), 12 μM of each primer, 2.5 μM of the probe, 1 μL of 10 pM dNTPs (Invitrogen, USA), and 1 U of DNA polymerase (Promega, USA); nuclease-free water (Invitrogen, USA) was added to achieve a final volume of 25 μL. Threshold cycle (Ct) values were defined as the cycle number at which the amplification curve crosses the fluorescence threshold set at 0.1 in the Rotor-Gene Q thermal cycler software (Qiagen, Germany). An increase in fluorescence intensity above this level, coupled to a cycle threshold of < 40, was considered a positive result. If a sample repeatedly tests inconclusive, it was considered positive. If a sample does not register a Ct value, it was considered negative. Each sample was run in triplicates to calculate standard deviation (SD). The sample was considered positive only when two or more technical replicates tested positive.

#### Specificity

Specificity was evaluated using BLAST and a panel of viruses listed in Table 1. The assay was tested in the presence of the target viral DNA (LSDV) as well as in the presence of non-target templates and in a mix of non-target background genomic DNA.

Titers for inclusivity and exclusivity for the target viral DNA ranged between 1.87 × 10^2^ and 2.55 × 10^3^ TCD50/mL and that for non-target templates were not lower than 1 × 10^5^ TCD50/mL to verify primers and probe specificity in an abundant non-target background genomic DNA.

#### Sensitivity evaluation

The limit of detection (LOD) of the PCR assay was determined using serially diluted genomic DNA of LSDV with a starting virus titer of 5.23 lg TCD50/cm^3^. LOD was defined as at least 95% positive replicates at the terminal dilution out of 20 replicates tested [11]. Five tenfold dilutions were initially prepared followed by three twofold serial dilutions. The slope was used to determine the reaction efficiency according to the equation: E = [10 (slope) − 1] × 100, where E = 100 corresponds to 100% efficiency. The repeatability and coefficient of variation (CV) were assessed by examining the same five tenfold dilutions (LSDV Dagestan/2015 [12], with a titer of 1.23 lg TCD50/cm^3^) in five repetitions on three different days. Statistic evaluation was conducted using Statistica v.10 (StatSoft, USA).

#### Clinical samples

A total of 243 clinical samples, which included 98 samples of serum, 118 of blood, and 34 of parenchymatous organs obtained from cows with clinical diseases in Russia from 2015 to 2017, were used to test the reliability of the developed assay.

### Results and discussion

**Fig. 2:**
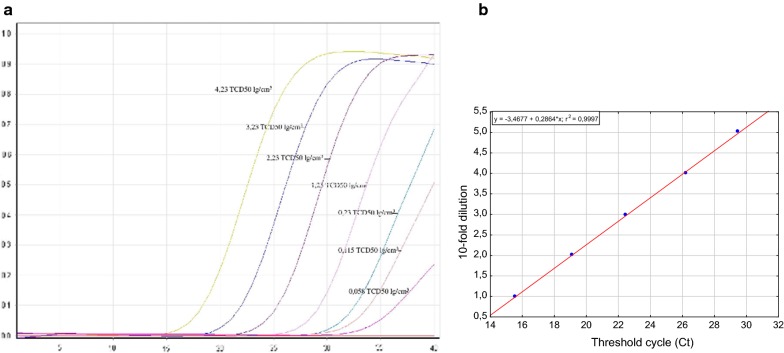
**a** Amplification curves obtained over five orders of magnitude. From the left to the right, the curves represent five tenfold serial dilutions (performed in triplicates) and three twofold dilutions (performed in 20 replicates), from 4.23 to 0.025 lg TCD50/mL, of DNA. **b** Linearity of the PCR assay results over five orders of magnitude. The equation of the standard curve obtained was y =  − 3.4677 + 0.2864*x, and the determination coefficient r2 was 0.9997. Reactions of the no-template controls tested showed no Ct values (data not shown)

The primers shared homology with other capripoxviruses based on BLAST hits, whereas the probe was unique to LSDV. Following testing against various field and reference capripoxvirus strains, the PCR was found to be highly specific (Table 1). In addition to the capripoxvirus strains listed in Table 1, the assay was evaluated against cow pox, orf, and peste de petite ruminants viruses as well and no false positive results were obtained (data not shown). To assess the reaction sensitivity, a series of five tenfold dilutions of viral DNA followed by three twofold dilutions from the last tenfold dilution were prepared and examined. The obtained preliminary results suggested that the assay’s detection limit was as low as 0.5 lg TCD50/cm^3^ (Fig. 2a). However, the subsequent testing of 20 replicates exhibited that the defined LOD corresponded to 0.115 lg TCD50/cm^3^ because the first twofold dilution generated 100% positive replicates with SD ± 0.72, whereas the second twofold provided only 80% positive replicates. The third twofold dilution—the terminal dilution (0.025 lg TCD50/cm^3^)—failed to generate any Ct value.

The obtained linearity results presented in Fig. 2 revealed that the amplification efficacy, determined over five orders of magnitude, was 93% with SD ranging from 0.11 to 0.23 (Fig. 2b). The repeatability was calculated by assessing the homogeneity among and within three replicates by the percentage of total variance obtained with five replicates of a single sample. An extremely low variation in CV values was obtained, indicating that the assay was highly repeatable. The standard deviation and CV ranged from 0.22 to 0.32 and 0.83% to 1.22%, respectively, over five repetitions across three runs.

A total of 243 clinical samples, including samples of serum, blood, and parenchymatous organs, were collected from LSDV-affected cows in Russia from 2015 to 2017. The universal assay showed 100% agreement with the P32-based capripoxvirus assay that was performed in parallel with the same panel of samples [13].

The present study describes a new screening PCR assay capable of detecting LSDV at the species level regardless of a genetic variant. There are few studies reporting a TaqMan real-time PCR assay that can detect LSDV DNA. Vidanovich et al. [14] reported a real-time PCR assay that used a TaqMan probe to detect the genome of LSDV field strains based on a 27-bp deletion in the EEV gene. The assay only tested positive with virulent field strains and did not positively identify live attenuated vaccine strains. However, considering the vaccination campaigns with Neethling-based live attenuated vaccines against LSDV launched in EU countries and Kazakhstan in 2016, a DIVA strategy based on the GPCR target was offered in a duplex format [10]. This strategy was able to distinguish between field and vaccine LSDV strains using a single target region shared by both strains. Surprisingly, a novel LSDV strain was recently identified that retained the backbone of vaccine LSDV strains with a patchwork of field LSDV DNA located throughout the genome [8]. The nature of the novel LSDV strain raises concerns and questions the genotype that it belongs to when detected in an affected cow in the field. When evaluated against the EEV target, it is detected as a non-field strain, whereas when evaluated against the GPCR target, it is detected as a vaccine strain, which is incorrect because the detected recombinant no longer appears to be the vaccine strain that was administered. Moreover, the detection of this virulent recombinant strain is not formally subject to the OIE notification, and the clinical picture could be misrepresented as “formally acceptable” despite the severity observed. Adverse effects of live vaccines against LSDV have previously been described [15, 16]. In this inconclusive scenario where the currently used PCR techniques are imperfect, an accurate polyvalent molecular tool for LSDV detection is required to perform diagnostics and undertake preventative measures. Therefore, the LSD044 targeting assay presented here serves as an easy screening technique for unambiguous diagnostics of lumpy skin disease. Despite the recombination, the targeted locus of the selected LSD044 was retained in the recombinant strain, which additionally argues for the successful selection of a conserved region for probe binding. Further investigations using the positive LSDV DNA samples can be supplemented by sequencing to clarify the genetic background of the strain, considering that the current DIVA assays can fail.

## Limitations

Although the present study is the first to develop a screening assay targeting all possible genetic variants of LSDV based on a conserved region, a panel of strains for validation only came from Russia, where naturally occurring recombinant stains were previously reported. To extend the range of application, additional samples from different geographical backgrounds are required.

## Data Availability

All data are presented within the manuscript.

## Abbreviations

bp: base pair
BHQ-1: Black Hole Quencher-1
Ct: threshold cycle
GTPV: goat pox virus
DIVA: differentiating infected from vaccinated animals
FAM: 6-carboxy fluorescein
LNA: Locked Nucleic Acid
LSDV: lumpy skin disease virus
SD: standard deviation
SPPV: sheep pox virus
